# Sparse discriminative latent characteristics for predicting cancer drug sensitivity from genomic features

**DOI:** 10.1371/journal.pcbi.1006743

**Published:** 2019-05-28

**Authors:** David A. Knowles, Gina Bouchard, Sylvia Plevritis

**Affiliations:** 1 Department of Radiology, Stanford University School of Medicine, Stanford, California, USA; 2 Department of Genetics, Stanford University School of Medicine, Stanford, California, USA; 3 Department of Biomedical Data Science, Stanford University School of Medicine, Stanford, California, USA; University of Cambridge, UNITED KINGDOM

## Abstract

Drug screening studies typically involve assaying the sensitivity of a range of cancer cell lines across an array of anti-cancer therapeutics. Alongside these sensitivity measurements high dimensional molecular characterizations of the cell lines are typically available, including gene expression, copy number variation and genomic mutations. We propose a sparse multitask regression model which learns discriminative latent characteristics that predict drug sensitivity and are associated with specific molecular features. We use ideas from Bayesian nonparametrics to automatically infer the appropriate number of these latent characteristics. The resulting analysis couples high predictive performance with interpretability since each latent characteristic involves a typically small set of drugs, cell lines and genomic features. Our model uncovers a number of drug-gene sensitivity associations missed by single gene analyses. We functionally validate one such novel association: that increased expression of the cell-cycle regulator C/EBP*δ* decreases sensitivity to the histone deacetylase (HDAC) inhibitor panobinostat.

## Author summary

A core tenant of precision medicine is that treatment should be tailored to the patient. In the context of cancer, large-scale screens, assaying the sensitivity of many cell-lines to panels of drugs, have the potential to enable discovery of biomarkers of sensitivity to specific therapeutics. However, existing computational approaches have not taken full advantage of these data. We develop a novel multi-task regression model, Lacrosse, which uses a Bayesian non-parametric prior to model “latent characteristics” of cell-lines that confer sensitivity to specific drugs and are predictable from genomic features. The resulting algorithm improves upon existing work by: a) jointly modeling multiple drugs to share statistical signal b) incorporating prior knowledge in terms of known inhibition targets c) using a sparse latent variable regression approach giving interpretable summaries of detected gene-drug associations. In particular, our analysis uncovers groups of drugs whose efficacy depends on genomic features in a similar way. We find new potential biomarkers of drug sensitivity, one of which we validate experimentally: that panobinostat is less effective when C/EBP*δ* is over-expressed.

## Introduction

Several drug screening studies have assayed the sensitivity of a library of cancer cell lines to an array of anti-cancer compounds. Notable examples are the Cancer Cell Line Encyclopedia [1, CCLE], Genomics of Drug Sensitivity in Cancer [2, 3] the 2012 DREAM challenge [4, 5] and the Cancer Therapeutics Response Portal v2 [6, CTRPv2]. Along with viability response curves, these studies provide high-dimensional molecular profiling of the assayed cell lines. For example, CCLE includes gene expression microarrays, copy number variation (CNV), and oncogene mutation status assays.

These data have the potential to both help understand the key differences between cancers and cancer subtypes that drive resistance to specific drugs, and to one day help choose the appropriate drug (or combination of drugs) for an individual patient, the core idea of precision medicine. As a result there is a need for analyses that both identify what differences between cancers cause the observed sensitivity patterns, and which differences can accurately predict what drugs will be efficacious for a tumor based on its genomic profile. The existing analyses of these datasets involve simple per drug regressions, such as elastic net [7]. While these methods are able to pick out the strongest signals in the data, they suffer from not taking advantage of known relationships between drugs and between genomic features. For example, we know which drugs have the same molecular target, and which features are related to the same gene, e.g. gene expression, CNV and mutation status will all typically be assayed for a given gene.

**Fig 1:**
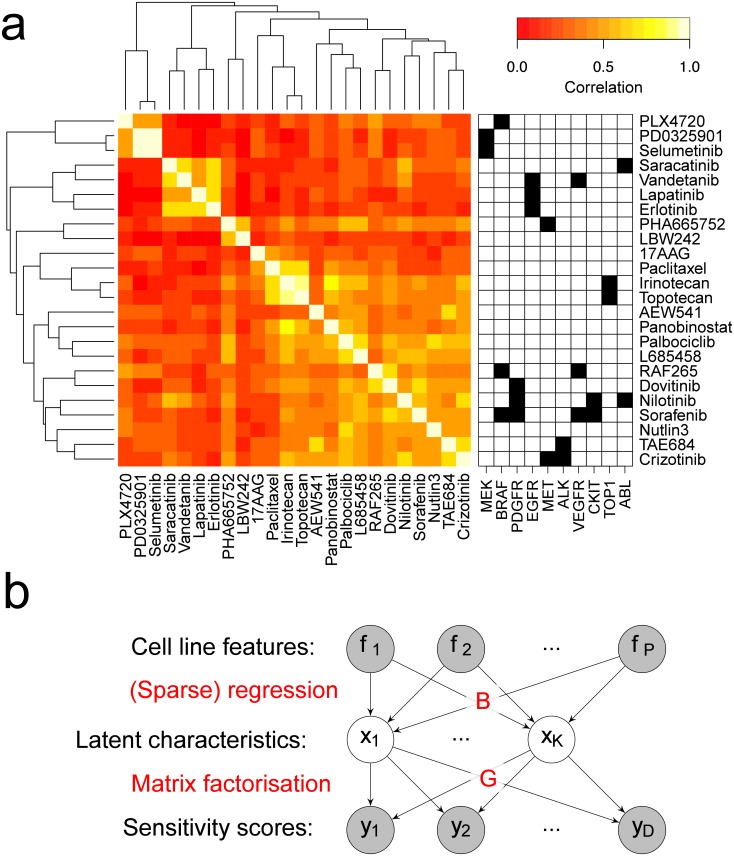
Joint analysis of cell line sensitivity across multiple drugs has the potential to improve predictive accuracy as well as model interpretability. **a**. While cancers are genetically heterogeneous there are phenotypic characteristics shared by many cancers subtypes, some of which are illustrated here. We refer to these as *latent* characteristics (LCs) because they are not directly observable from genomic data, but we hypothesize that each characteristic will have a detectable genetic signature and a defined influence on drug sensitivity. **b**. Pearson correlation of drug sensitivity profiles *(active area scores)* across CCLE, annotated by known inhibition targets. **c** The Lacrosse model consists of two components, shown here graphically. The first is a sparse linear regression from cell line features, **F** (gene expression, copy number variation and genomic mutations) to continuous valued latent characteristics, **X**. The second is a sparse factor analysis (matrix factorization) where the latent characteristics (the factors) explain the observed sensitivity scores through a sparse loading matrix **G**.

Cancer is highly heterogeneous in terms of its genomic features but many cancers share common phenotypic characteristics (Fig 1a), the “hallmarks of cancer” [8]: broken apoptosis or cell cycle regulation [9], disrupted DNA repair mechanisms [10], or “addiction” to specific oncogene pathways [11]. Because these phenotypic features can not be directly observed from genomic data, we regard them as unobserved, latent characteristics in this work. We expect these unobserved, latent characteristics to be associated with genomic cell line features such as gene expression. Moreover, we expect that the presence or absence of these latent characteristics confers sensitivity or resistance to specific therapeutic compounds.

To predict cancer drug sensitivity based on latent characteristics derived from genomic-drug screening data, we propose a novel approach, LAtent ChaRacteristics Of Small-molecule SEnsitivity (Lacrosse). The statistical model underlying Lacrosse is a discriminative, Bayesian non-parametric, sparse factor analysis, which falls under the general class of multitask regression models [12]. A handful of related statistical methods have been used for drug sensitivity prediction. Menden et al. [13] used a single hidden layer feed-forward neural network [14]. While multitask neural networks are easily constructed by allowing multiple outputs to share the same hidden layers, Menden et al. took the alternative approach of featurizing the drugs using the chemoinformatic program PaDEL [15] and learning a single predictive model learned using both the drug and cell lines features. In contrast, Lacrosse does not featurize the drugs.

Lacrosse is closely related to kernelized Bayesian multitask learning [16, KBMTL]. While their approach gives excellent predictive performance, interpretability is somewhat lacking since the kernels (similarity between cell lines) are calculated on entire “views” (e.g. continuous gene expression) so that the influence of specific genes or pathways is not elucidated. We compare to KBMTL here and show comparable, or even slightly improved predictive performance, with the added benefit of increased interpretability.

A Bayesian multitask multiview linear regression (MVLR) was recently developed [17]. Sparse Cauchy priors are used to select features and a Dirichlet prior is used over a parameter vector that selects predictive views. While the approach is multitask the relationship between a feature and drug response is the same for all drugs included in the model, up to a positive multiplicative weight. This is analogous to Lacrosse restricted to only one latent characteristic.

A distinct approach is taken by Knijnenburg et al. [18] who develop ‘Logic Optimization for Binary Input to Continuous Output’ (LOBICO) which finds small, interpretable logic networks predictive of drug response for individual drugs. LOBICO is limited to binary cell-line features.

Two other recent studies have developed analyses that share ideas with Lacrosse but which provide exploratory analyses rather than predictive models of drug response. This makes it infeasible to compare to our approach in terms of predictive performance. First, Seashore-Ludlow et al. [6] developed Annotated Cluster Multidimensional Enrichment (ACME), which tests whether drug/cell line sensitivity biclusters have coherent biological signal in terms of enriched protein targets (for the drugs) and mutations/lineage (for the cell lines). Second, El-Hachem et al. [19] applied Similarity Network Fusion [20] to drug-drug networks derived similarity in terms of chemical structure, drug sensitivity, and drug perturbation response.

Lacrosse also has similarities to nuclear norm regression [21], an extension of L1-regularized regression [7] to the multitask setting by penalizing the trace/nuclear-norm (the sum of singular values) of the coefficient matrix. Compared to their optimization based approach, DNFSA, as a Bayesian probabilistic approach has the advantage of allowing incorporation of prior knowledge in the form of known drug-drug relationships.

An additional unique aspect of Lacrosse is that it allows a graph over the drugs to be specified encoding prior knowledge about which drugs are likely to have similar properties. This graph is used to specify a Markov-random field which explicitly encourages drugs which share edges to have more similar coefficients in the model.

## Results

### Summarizing dose-response curves using active area

Large-scale viability screens such as CCLE have typically summarized dose-response curves in terms of the drug concentration required for 50% inhibition of growth, “IC50”. IC50 however has several weaknesses: a) it is undefined if 50% inhibition is never reached, b) it is noisy due to being overly reliant on viability measurements close to the IC50 value, and c) it ignores differences in effectiveness for doses above the IC50 point. An simple alternative summary is “active area”, the integrated area above the dose-response curve (S1 Fig). We have found active area to be more predictable from molecular profiles than IC50: 10-fold cross-validation on CCLE using group LASSO explains 27.5% of heldout variance in active area scores across drugs, compared to only 14.4% for IC50. We therefore choose to use active area as the drug sensitivity metric throughout this work.

### Known drug targets only partially explain sensitivity profiles

For the 24 drugs in CCLE we analyzed the between-drug correlation of sensitivity profiles (active area scores) across 432 cell lines (Fig 1b). In some cases the high correlation between profiles is explained in terms of shared inhibition target: e.g. the MEK inhibitors PD0325901 and selumetinib have a highly similar sensitivity profile across cell lines. Similar statements can be made for inhibitors of EGFR (erlotinib, lapatinib, vandetanib), TOP1 (topotecan, irinotecan) and ALK (crizotinib, TAE684). However, in other cases are less easily explained: PHA665752, a c-MET inhibitor, and LBW242, a SMAC mimic, have significantly correlated profiles but no known shared mechanism of action. These observations motivate using known inhibition targets as soft prior knowledge rather than hard constraints in our methodology.

### Lacrosse overview

Lacrosse is a Bayesian-nonparametric, sparse, multitask regression that jointly predicts viability for multiple drugs using cell line genomic features. Lacrosse posits that subsets of cancer cell lines possess latent characteristics (LCs) which are predictive of their sensitivity profile across different drugs. Whether a cell line possesses a particular LC is identified by the presence of genomic features specific to the LC, that is, the LCs are generated by a sparse regression on the cell line features.

Explicitly, given the matrix **F** of genomic features (# features × # cell lines), Lacrosse models the matrix **Y** of viability scores (# drugs × # cell lines) as

$$\begin{array}{c} Y\approx GX,\;X\approx BF, \end{array}$$ (1)

where **X** are the LCs, and **G** and **B** are matrices of (sparse) regression coefficients to be learned (Fig 1c). We use 40,492 genomic features from CCLE spanning gene expression, CNV and mutations. These LCs represent a low dimensional embedding of the cell lines which preserves information salient to their drug sensitivity profiles. Using this graphical model the LCs are primarily focused on modeling the drug sensitivity patterns, but are also constrained to be predictable from cell line genomic features.

**Fig 2:**
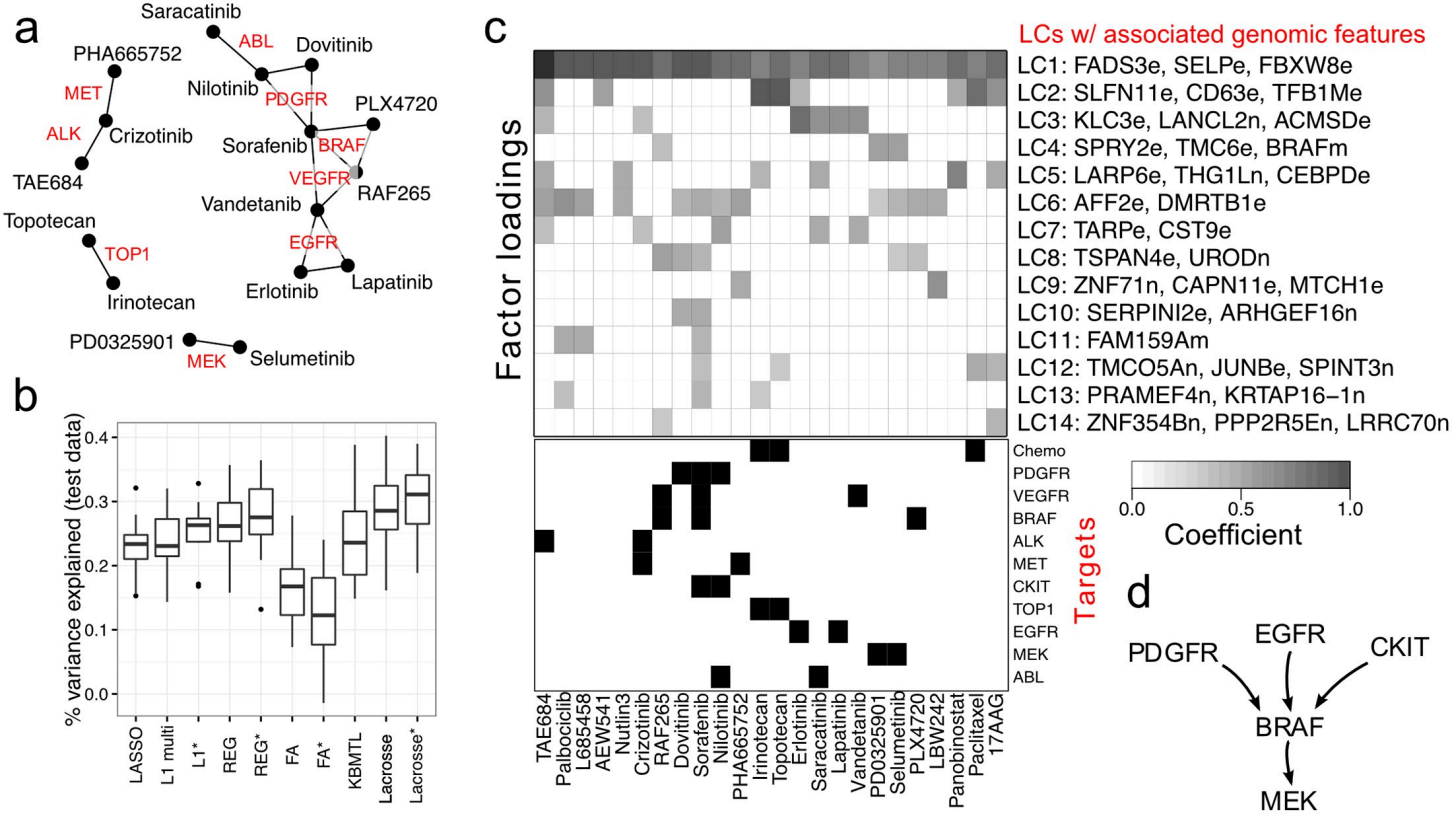
Lacrosse combines prior knowledge (shared inhibition targets) and observed similarity between drugs to improve predictive performance and define overlapping clusters of drugs with related dependence on genomic features. **a**. A Markov random field (MRF) is used to encode prior knowledge of which drugs share inhibition targets. Lacrosse does not use the inhibition targets themselves, only that if two drugs share an inhibition target then they are more likely to share sensitivity to a particular latent characteristic. **b**. Predictive performance results on CCLE. Here LASSO = L1 regression, REG = spike & slab regression. Lacrosse = discriminative factor analysis. In general incorporating prior knowledge about between drug relationships (the methods denoted with *) improves predictive performance. The Bayesian spike and slab regression based methods (REG and Lacrosse) also perform somewhat better than the L1 optimization method (LASSO, L1 multi, and L1*), although this comes at considerable computational expense. The factor analysis models (FA and FA*) have quite poor performance, likely to due to not being discriminative. The performance of KBMTL is similar to that of LASSO. **c**. Lacrosse* factor loadings: 14 latent characteristics (LCs) × 24 drugs, known targets for each drug, and associated gene features (i.e. non-zero coefficients in **B**) for each LC. Encoding of gene features: e = expression, n = copy number variation, m = mutation. **d**. A highly simplified layout of the MAPK pathway associated with latent characteristic 4.

We additionally extend Lacrosse to allow prior knowledge about both drugs and genomic features to be incorporated in the form of a graph where related drugs (or genomic features) share edges. Connected nodes are encouraged to have the same regression coefficient sparsity pattern using a Markov-random field (MRF) approach. In practice we use this capability to inform the model about which drugs share molecular targets (hand-curated, Fig 2a) and which cell line features correspond to the same gene.

### Predictive performance

To assess the predictive performance of Lacrosse compared to existing state-of-the-art methods we performed 10-fold cross-validation holding out 10% of cell lines for each fold and calculated the proportion of variance explained (PVE) for each fold. We compared Lacrosse to

- FA: sparse, nonparametric factor analysis jointly over the molecular characteristics and sensitivity [22]
- REG: spike and slab Bayesian linear regression [23]
- L1: LASSO L1-regularized linear regression [24] using the glmnet R package
- L1 multi: multiresponse regression using group LASSO [25] resulting in a shared sparsity pattern across all drugs, again using glmnet
- KBMTL: kernelized Bayesian multitask learning [16].
- Ridge: ridge regression.
- Elastic Net: with $\alpha =\frac{1}{2}$
- Bayesian multitask multiview linear regression (MVLR) [17]

For each of these approaches we additionally consider an MRF extension (see Methods) which encourages drugs with shared targets to have similar sets of coefficients with non-zero weights. For LASSO regression we analogously enforce that any drugs sharing an inhibition target have the same sparsity pattern. These extensions are denoted with a superscript asterisk (*) on the method acronym.

Lacrosse outperforms these baselines in terms of its ability to predict sensitivity across drugs for heldout cell lines in CCLE (see Fig 2b). The sparse factor analysis (FA) approaches perform poorly, presumably because so much modeling capacity is effectively wasted in modeling the thousands of genomic features that are not be associated with sensitivity. Of the regression based approaches LASSO, group LASSO and KBMTL perform comparably with PVE around 24%. Bayesian spike-and-slab sparse regression (REG) somewhat outperforms these methods, with PVE = 26%. Finally Lacrosse, being able to share statistical signal across drugs, performs best, with PVE = 28.5%. In all cases apart from FA, we additionally find that explicitly incorporating known relationships between drugs (in terms of shared inhibition targets) and genomic features (corresponding to the same gene) improves predictive performance. Performance for FA may have dropped using known relationships since the model capacity is already insufficient to accurately associate genomic features with sensitivity, and added constraints further reduce the effective model capacity. Lacrosse including the MRF-extension achieves a median PVE across folds of 31.5%.

**Fig 3:**
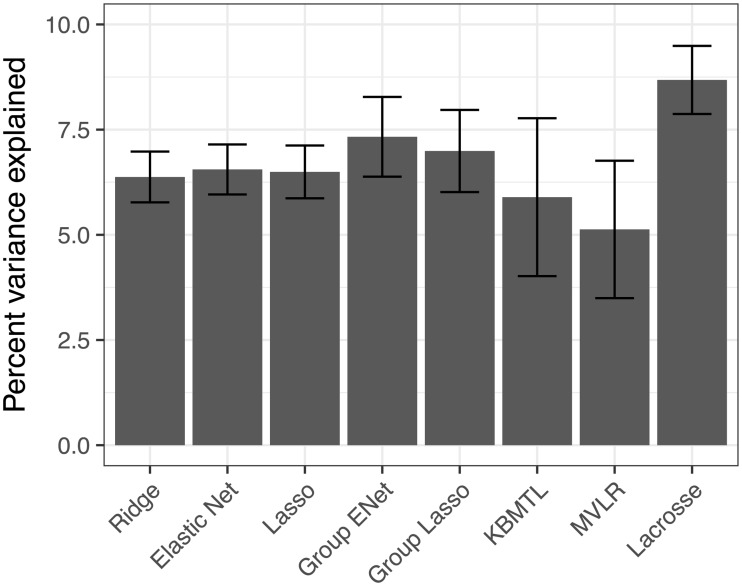
Cross-validated predictive performance on the large CTRPv2 sensitivity screen. Lacrosse significantly outperforms Group Lasso and Elastic Net, which themselves surpass the single task methods as well as KBMTL and MVLR.

We additionally assessed predictive performance in terms of PVE on the 545 drugs, 783 cell lines CTRPv2 dataset (Fig 3), again using 10-fold cross-validation. The three tested single task methods—ridge regression, Elastic Net and LASSO—all performed similarly. Group LASSO outperforms LASSO (*p* = 0.032, paired t-test), and is itself outperformed by Group Elastic Net (*p* = 0.0028, paired t-test). In our hands KBMTL underperforms Group LASSO (*p* = 0.036) and Group Elastic Net (*p* = 0.005) and has comparable performance to the single task methods. MVLR significantly underperforms the single task methods (*p* = 0.007, 0.005, 0.006 for ridge, LASSO and Elastic Net respectively) although we emphasize that MVLR is only designed for joint modeling of closely related drugs (e.g. those with shared inhibition targets) so it is unsurprisingly it performs poorly at simultaneous modeling of over 500 drugs with many different mechanisms-of-action. Finally Lacrosse outperforms Group Elastic Net by a significant margin (*p* = 0.0008, paired t-test). The PVE across folds was not found to be significantly non-normal by a Shapiro-Wilk test for any method, but we none-the-less confirmed all comparisons found significant by t-test were also significant by Wilcoxon signed rank test, apart from KBMTL outperforming Group LASSO. We confirmed the qualitative ordering of the methods held up when assessing performance using the concordance index (S2 Fig). While Lacrosse obtains a higher mean c-index than Group Elastic Net (0.613 vs 0.608) this difference is not statistically significant.

We varied Lacrosse’s MRF strength parameter and found performance to be robust within a wide range of values (S3 Fig). The 29 LCs discovered running on the full CTRPv2 dataset are available here: https://www.dropbox.com/s/cucayg3nulkikjb/CTRPv2_LCs.zip?dl=0.

Comparison with LOBICO [18] is complicated by the fact that it operates on binary features (typically mutations) and response (binarized AUC or IC50) (the continuous response is used to weight samples however). Considering this we compared LOBICO to single-task LASSO either using continuous AUC as response, or L1-regularized logistic regression using the same binarization as for LOBICO. For all three methods we used mutation data only (for 1600 genes) to accommodate LOBICO’s requirement for binary features. Across 11 randomly chosen drugs from CTRPv2 LOBICO is substantially and consistently outperformed by both L1-regression based models (S4 Fig). We hypothesize that while LOBICO can explore a small hypothesis space of genes (<100) known to be important in conferring sensitivity or resistance, it is unable to effectively screen a larger number of potentially relevant features. While Iorio et al. [3] were able to build significantly predictive and interpretable LOBICO models with > 600 features, they did not compare predictive performance to sparse regression methods.

We explored whether the predictive model learnt on CTRPv2 would generalize to independent data. We first assessed generalization performance in CCLE (S5 Fig). For all 14 drugs common to both datasets we see statistically significant prediction (Benjamini-Hochberg adjusted Spearman correlation between predicted and observed AUC *p* < 0.004), and qualitatively the concordance indices are comparable to in-sample accuracy assessed using pre-validation in CTRPv2, with the exceptions of Nutlin-3 and RAF265. Since CTRPv2 includes all cell lines assayed in CCLE we next assayed generalization in The Genentech Cell Line Screening Initiative (gCSI) [26]. 37 cell lines were profiled in gCSI that were not in CTRPv2. We show results both for all cell lines in gCSI and the 37 non-overlapping cell lines alone in S6 Fig. Of the 10 drugs in common between CTRPv2 and gCSI, 7 are statistically significantly predicted on the full gCSI cell line collection (Benjamini-Hochberg adjusted Spearman correlation *p* < 0.05), but MS-275, Bortezomib and Crizotinib are not. On the 37 shared cell lines only Vorinostat shows significant prediction, but this maybe due to limited power with only *n* = 37 test points. Finally we applied the CTRPv2 model in GDSC1000 [3] for 69 shared drugs. Across all *n* = 1110 cell lines in GDSC1000, 59 drugs showed significant prediction, compared to 50/69 when using the *n* = 501 non-overlapping cell lines (Benjamini-Hochberg adjusted Spearman correlation *p* < 0.05, S7 Fig).

### Drug-gene associations

**Fig 4:**
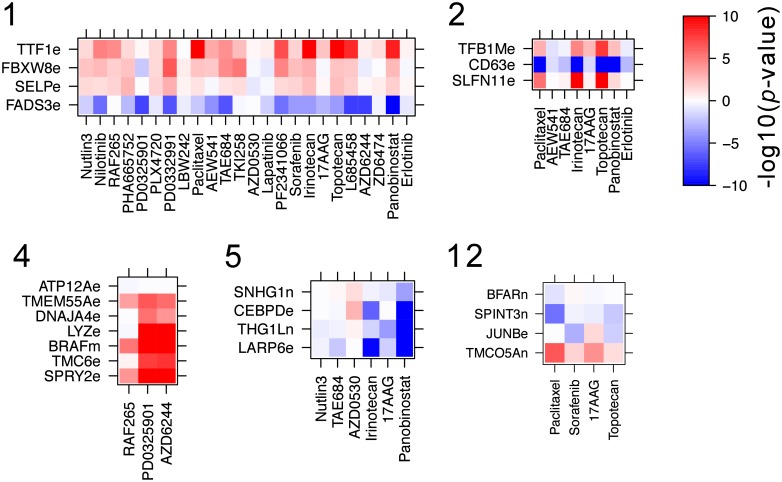
A subset of the learned latent characteristics represented as clusters of genomic features (rows) and drugs (columns). Numbering corresponds to the internal LC identifier and is arbitrary. Colors represent −log_10_ *p* for the relationship between the feature and drug, with the sign representing positive or negative associations (using Spearman correlation). Genomic feature suffixes e = expression, n = copy number variation, m = mutation. The remaining LCs are shown in S8 Fig.

Analysis of the latent characteristics can provide insights into signaling and regulatory pathways predictive of drug response. We tested pairwise relationships between the drugs and genomic features in each LC from CCLE using Spearman correlation, with selected LCs of interest shown in Fig 4. Remaining LCs are shown in S8 Fig. While many strong associations are seen, other associations are weaker, suggesting that the relationships uncovered by Lacrosse rely on sharing statistical power across drugs within an LC. LC 1 has positive loading for all drugs, suggesting there is some shared behavior across all the drugs in this dataset, a phenomenon previously described as “general levels of drug sensitivity” (GLDS) [27]. Indeed, LC 1 is highly correlated with the first GLDS (*R*^2^ = 0.75, *p* < 2 × 10^−16^, S9 Fig). The expression level of FBXW8 is a genomic feature of LC 1, which is interesting since this gene is known to play an essential role in cancer cell proliferation [28]. This association was not picked up by the per drug regression, presumably because Lacrosse gains statistical power by analyzing all the drugs simultaneously.

Some LCs include known related drugs, e.g. LC 2 involves the DNA-damaging agents irinotecan and topotecan, as well as another chemotherapeutic agent, paclitaxel. It is reassuring to see SLFN11 expression modulating sensitivity to irinotecan and topotecan in this LC, since this is a known and experimentally validated relationship [29]. Increased CD63 expression decreases sensitivity to the drugs in LC 2, particularly paclitaxel, irinotecan, topotecan and panobinostat. While this association is novel to the best of our knowledge, CD63 is known to be an exosomal marker that correlates with invasiveness in ovarian cancer cell lines [30].

LC 4 includes mutation of B-RAF as a genomic feature and drives sensitivity to the B-RAF inhibitor RAF265 and MEK inhibitors (PD0325901 and selumetinib/AZD6244). As a result, we conclude LC 4 corresponds to the MAPK pathway, a signaling cascade involving B-RAF, MEK and ERK (see Fig 2d). Since Lacrosse builds a sparse predictive model so may exclude some meaningful associations. Indeed we find LC4 level is correlated with B-RAF (*p* = 6 × 10^−20^) and KIT (*p* = 0.016) mutations, PDGFRB expression (*p* = 1 × 10^−4^) and MAP2K1 copy number (*p* = 0.03). 86/253 (34%) genes in the KEGG MAPK pathway are significantly correlated with LC4 level across cell lines, a 1.5× enrichment compared to background (Fisher’s exact *p* = 0.002). Other interesting associations in LC 4 are those with TMC6 and SPRY2. SPRY2 is downstream of the MAPK pathway, but SPRY2 inhibition also activates MAPK and can lead to tumorigenesis [31], suggesting an as yet poorly characterized feedback loop. While TMC6 is not known to be involved in MAPK signaling, there are suggestive hints: TMC6/8 forms a complex with ZnT-1 [32], a zinc-finger protein which itself activates the MAPK pathway [33]. Potentially also of interest is that nonsense mutations in TMC6 cause a hereditary condition called Epidermodysplasia verruciformis involving susceptibility to human papillomavirus and resulting in cutaneous squamous cell carcinomas [32].

The influence of JunB on the VEGF inhibitor sorafenib and heat shock protein 90 (HSP90) inhibitor tanespimycin in LC 12 is likely due to regulation of the VEGF pathway by JunB [34], and the fact that HSP90 is required for VEGF induction. Finally we note that hypermethylation of TMCO5A is associated with worse prognosis in ovarian cancer [35].

### Functional validation

In LC 5 we observe that sensitivity to the HDAC-inhibitor panobinostat is associated with C/EBP*δ*, a transcription factor that regulates cell cycle progression and apoptotis, and is therefore a putative tumor suppressor [36]. While C/EBP*δ* has not previously been associated with drug sensitivity, high expression is known be associated with poor prognosis across glioblastomas [37] and the closely related C/EBP*β* is recognized as a synergistic master regulator with STAT3 of the mesenchymal phenotype in aggressive glioma [38]. Since this association was not reported using single drug analyses of the CCLE dataset and because of the significant current interest in HDAC-inhibitors, we aimed to functionally validate this finding. The association between C/EBP*δ* mRNA expression and sensitivity to panobinostat is replicated in LC 2 from CTRPv2, with the pairwise relationship also being highly significant (Spearman *ρ* = −0.30, *p* < 1 × 10^−15^).

**Fig 5:**
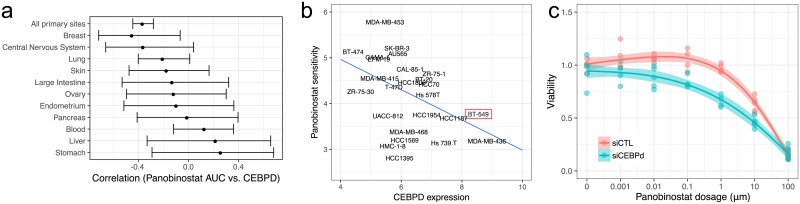
Knock-down of the cell-cycle regulator C/EBP*δ* increases sensitivity to the HDAC-inhibitor panobinostat *in vitro*. **a**. The negative association between C/EBP*δ* expression and sensitivity to panobinostat is seen across a range of cancers, but is most pronounced in breast cancer cell lines. Whiskers denote 95% confidence intervals. **b**. Within breast cancer cell-lines we chose to use BT-549 since it has relatively high C/EBP*δ* expression and low panobinostat sensitivity. **c**. siRNA mediated knock-down of C/EBP*δ* increases the sensitivity of BT-549 cells to panobinostat.

The relationship between C/EBP*δ* expression and sensitivity to panobinostat is strongest in breast cancer cell lines (Fig 5a). Of these, we chose the invasive ductal carcinoma cell line BT-549 as our model system, since BT-549 expresses relatively high levels of C/EBP*δ* and has low sensitivity to panobinostat (Fig 5b), allowing us to effectively test the hypothesis that knocking down C/EBP*δ* will increase panobinostat sensitivity. We treated BT-549 cells overnight with either a short interfering (si) RNA targeting C/EBP*δ* or a control non-targeting siRNA. We confirmed cells remained viable after C/EBP*δ* knock-down (S10a Fig) and that the knock-down was successful by Western blot (S10b Fig). Cells were then incubated for 24h at different panobinostat concentrations. The C/EBP*δ* knock-down (KD) increases sensitivity to panobinostat: a reduction in viability to 66% is detectable for the KD condition at a drug concentration of 1*μ*M, whereas for the control cells viability remains at 97% (Fig 5c). At 10*μ*M viability is reduced to 46% and 63% for the KD and control respectively. Using 4-parameter log-logistic growth curves estimated using the drc R package [39] IC50 values are 5.1*μ*M (KD) and 17.0*μ*M (control). Based on a ANOVA comparing one vs. two cubic spline fits, the difference in these response curves is statistically significant (*p* = 0.03).

## Discussion

We introduced Lacrosse, a model capable of learning latent characteristics which explain observed sensitivity of cancer cell lines to groups of related drugs, and which are predictable from genomic features. Using a Bayesian nonparametric approach Lacrosse is able to adaptively learn an appropriate number of latent characteristics from data. Compared to kernel methods such as KBMTL, Lacrosse’s underlying sparse regression allows straightforward interpretation via visual inspection of the small number of LCs. Lacrosse allows straightforward incorporation of “soft” prior knowledge in the form of a graph over drugs representing known drug-drug relationships (shared inhibition targets in our analysis). Including such prior information in a non-Bayesian method such as nuclear norm regression would be challenging. Lacrosse uncovers both known and novel associations, one of which we were able to validate experimentally. Our finding that reducing C/EBP*δ* expression increases sensitivity to panobinostat suggests a therapeutic avenue if C/EBP*δ* expression could be reduced *in vivo* using a similar siRNA approach or alternative targeted methods such as anti-sense oligonucleotides [40].

Our positive functional validation result is encouraging, but there are limitations of our analysis. Genomic features, especially gene expression and CNV, have high levels of correlation so it is difficult to say which out of set of correlated features is most likely to be causally related to sensitivity. However, correlation is less problematic if we are primarily interested in the model’s ability to predict sensitivity, a valuable task in its own right due to its potential application in precision medicine. Lacrosse is relatively computational intensive, taking around 10h to complete 10,000 Gibbs sampling iterations on a modern workstation, compared to only around 20min for group Lasso (including cross-validation) on the same data. However, any future clinical application would not require retraining the model, only performing prediction, which is extremely fast once the model is trained. We experimented with running Lacrosse on subsets of cell-lines corresponding to specific cancer types, but found the reduced sample size resulted in substantially reduced predictive performance and a smaller number of LCs. One interesting future direction would be to extend Lacrosse to be multitask over not only drugs but also cancer types, allowing sharing of statistical strength across all cell lines but allowing tissue-of-origin specific associations when supported by the data.

We focused here on the CCLE and CTRPv2 dataset due to their dense coverage of drug-cell line pairs. In principle it would be beneficial to integrate additional large scale drug viability assays such as GDSC [3]. However, the agreement between these screens is modest due to the use of different growth assays and other technical variation [41] which would make such an analysis extremely daunting. CCLE does not include any epigenomic features such as methylation so we were not able to incorporate this into the model. However, it would be straightforward to include such data if it were available for a different dataset.

An intriguing direction is to utilize more features of the drugs than just known inhibition targets. For example, Menden et al. [13] showed promising performance summarizing the chemical structure of each drug as a feature vector using PaDEL-Descriptor [15], a popular chemo-informatics tool. An alternative would be to leverage a graph kernel like Weisfeiler-Lehman [42]. This avenue opens the exciting possibility of generalizing not only to new cell lines but also to new drugs.

## Methods

### Data acquisition and pre-processing

Cell line molecular profiles and drug sensitivity scores were obtained from the CCLE data portal. For mRNA expression gene-centric RMA-normalized values were log and then *z*-transformed (analysis date 2012-09-29). Mutations in 1651 genes determined by hybrid capture sequencing were summarized to a binary variable for each assayed gene denoting 1 for any nonsynonymous change and 0 otherwise (analysis date 2012-05-07). We used the CCLE recommended data where variants that are a) common polymorphisms, b) have allelic fraction <10%, c) are putative neutral variants or d) are located outside of the CDS for all transcripts are removed. Per gene DNA copy number as determined using Affymetrix SNP6.0 arrays were obtained (analysis date 2013-12-03). Pharmacologic profiles were taken from *CCLE_NP24.2009_Drug_data_2015.02.24.csv*, specifically columns “IC50” and “ActArea”.

For CTRPv2 data was obtained using PharmacoGx [43]. The mRNA expression, copy number and mutation data is the same as for CCLE and were pre-processed analogously. PharmacoGx recomputed active area scores were used as the response.

### The Indian buffet process

The Indian buffet process [44, IBP] defines a distribution over infinite binary matrices, which can be used to construct latent feature models where the number of features is unbounded a priori. Models constructed using the IBP are sparse in that only a small number of features are typically active for each entity. The IBP is the infinite limit of the finite *K* model,

$$\begin{array}{c} v_{k}|\alpha ∼\text{Beta}(\frac{\alpha }{K},1) \end{array}$$ (2)

$$\begin{array}{c} Z_{dk}|v_{k}∼\text{Bernoulli}(v_{k}) \end{array}$$ (3)

Taking the limit *K* → ∞, and rearranging columns of **Z** carefully, we obtain a stochastic process most easily described by a culinary metaphor. Consider a buffet with a seemingly infinite number of dishes (latent factors/LCs) arranged in a line. The first customer (drugs) starts at the left and samples Poisson(*α*) dishes. The *d*th customer (drug) moves from left to right sampling dishes with probability $\frac{m_{k}}{d}$ where *m*_*k*_ is the number of customers to have previously sampled dish *k*. Having reached the end of the previously sampled dishes, he tries $\text{Poisson}(\frac{\alpha }{d})$ new dishes. Element *Z*_*dk*_ of the *D* × *K* binary feature allocation matrix **Z** is 1 if and only if customer *d* tried dish *k*. In Lacrosse element *Z*_*dk*_ corresponds to whether drug *d* is influenced by LC *k*.

### Statistical model

In the Lacrosse generative process the Indian buffet process matrix, **Z**, determines which elements of a factor loadings matrix, **G** are non-zero. **G** is then used in the model,

$$\begin{array}{c} \overset{\left[D\times N\right]}{Y}=\overset{\left[D\times K\right]}{G}\overset{\left[K\times N\right]}{X}+\epsilon ,\;\overset{\left[K\times N\right]}{X}=\overset{\left[K\times P\right]}{B}\overset{\left[P\times N\right]}{F}+w, \end{array}$$

where *ϵ*, *w* represent noise, **Y** are the sensitivity measurements, **X** are the latent factors, **B** are regression coefficients, and **F** are the genomic features. For our application *D* is the number of drugs, *N* is the number of cell lines and *P* is the number of molecular features, including gene expression, CNV and mutations. Here *K* is the number of latent factors. The model is closely related to reduced rank regression, with the addition of the noise *w* on **X**.

We use a spike and slab prior on elements of **G**:

$$\begin{array}{c} G_{dk}|Z_{dk}∼Z_{dk}N\left(0,1\right)+\left(1-Z_{dk}\right)\delta_{0} \end{array}$$ (4)

where *Z*_*dk*_ ∈ {0, 1} indicates whether *G*_*dk*_ is non-zero, N is the Gaussian distribution, and *δ*_0_ is a delta point mass at 0. By using the Indian buffet process as a prior on **Z** we allow the model an unbounded number of latent characteristics *K*.

We also use a spike and slab prior on **B**,

$$\begin{array}{cc} B_{kp}|V_{kp} & ∼V_{kp}N\left(0,1\right)+\left(1-V_{kp}\right)\delta_{0},\\ V_{kp} & ∼\text{Bernoulli}\left(\pi_{p}\right)\;\pi_{p}∼\text{Beta}\left(\beta /P,1\right) \end{array}$$ (5)

We know that the sensitivity profile for some drugs is more easily predicted than others, so we use diagonal rather than spherical (isotropic) noise on **Y**, specifically the hierarchical prior

$$\begin{array}{cc} \epsilon_{dn}|\lambda^{e} & ∼N(0,1/\lambda_{d}^{e}),\\ \lambda_{d}^{e}|b & ∼G(1,1/b),\\ b & ∼G(1,1) \end{array}$$ (6)

where *G* is the Gamma distribution. We use an analogous prior on the noise for **X**, denoted *w*, since some latent characteristics may be better predicted by the molecular characteristics than others.

Inference is performed using 10,000 iterations of standard Gibbs sampling [45] implemented in C++ using Eigen (eigen.tuxfamily.org) and interfaced to R using RCpp/RCppEigen [46].

### Markov random field extension

The relationships between drugs and between features are easily represented by a Markov random field (MRF). For example, two drugs sharing a common target molecule are linked in the MRF, and two any features such CNV and gene expression for the *same* gene will have an edge between them. Following [47] we modify the IBP probability (see Eq 2) of a column *Z*_:*k*_ to be

$$\begin{array}{c} P(Z_{:k}|\tau_{k})\propto \underset{\text{MRF}\;\text{term}}{\underset{︸}{\text{exp}\;(\sum_{d^{\prime }<d}w_{d^{\prime }d}Z_{d^{\prime }k}Z_{dk})}}\underset{\text{usual}\;\text{IBP}\;\text{term}}{\underset{︸}{\prod_{d}v_{k}^{Z_{dk}}{\left(1-v_{k}\right)}^{1-Z_{dk}}}} \end{array}$$ (7)

where *w*_*d*′*d*_ is the edge weight between drugs *d*′ and *d* in the MRF. We use an analogous prior on *V*_*kp*_ to couple the probability of having non-zero coefficients in **B** for different features associated with the same gene.

### Cell culture and siRNA transfections

Human breast carcinoma cell line BT-549 was obtained from American Type Culture Collection (Manassas, VA) and cultured in RPMI-1640 medium, 2mM L-Glutamine (Gibco, ThermoFisher Scientific, Waltham, MA), supplemented with 10% FBS (Gibco, ThermoFisher Scientific, Waltham, MA), and 0.023 U/ml (BT-549) in a humidified atmosphere with 5% CO_2_ at 37°C.

For CCAAT/enhancer binding protein delta (C/EBP*δ*) silencing, cells were seeded (150 000 into 6-well plate or 10 000 cells into 96-well plate) and grown overnight prior to transfection. Cells were transfected using a non-targeting *Silencer* Select Negative Control siRNA (4390843, ThermoFisher Scientific, Waltham, MA) or siRNA targeting C/EBP*δ* (s2895, 4392420, ThermoFisher Scientific, Waltham, MA) using Lipofectamine 2000 (Invitrogen, ThermoFisher Scientific, Waltham, MA) according to the manufacturer protocol. Reagents were diluted in Opti-MEM reduced serum medium (Gibco, ThermoFisher Scientific, Waltham, MA) and transfection complexes were added to the cells at a final concentration of 20 nM. Transfection media was replaced with 10% FBS antibiotic-free RPMI with panobinostat (range of concentration from 0.01*μ*M to 10*μ*M) after overnight incubation with siRNAs and incubated for 24 h. Cells were harvested for assessment of knock-down efficiency using Western blot analysis or viability using RealTime-Glo MT Cell Viability Assay (Promega, Madison, WI) according to the manufacturer protocol.

### Western blot

Cell protein extracts were prepared with lysis buffer containing 50 mM Tris pH 8, 2% sodium dodecyl sulphate (SDS), 5 mM Ethylenediaminetetraacetic acid (EDTA), 5 mM Ethylene glycol-bis (2-aminoethylether)-N,N,N’,N’-tetraacetic acid (EGTA), 25 mM sodium fluoride (NaF) and 1 mM sodium orthovanadate (Na_3_VO_4_) supplemented with the protein inhibitor cocktail Complete Mini, EDTA-free (Roche, Indianapolis, IN). Lysates were briefly sonicated, vortexed, incubated 5 min at 4°C and vortexed again. Cellular debris were cleared by centrifugation at 12 000 rpm during 10 min. Supernatants were aliquoted and stored at -80°C for further use. Protein quantification assay was performed using a BCA Protein Assay kit (Pierce, ThermoFisher Scientific, Waltham, MA). The protein extracts (15 *μ*g) were applied on a 12% polyacrylamide-SDS gel electrophoresed at 200V during 45 min and transferred to a Immobilon transfer membrane (EMD Millipore, Billerica, MA) using the Mini Trans-Blot Cell (Bio-Rad, Hercules, CA) settled at 160V for 1 h. The membrane was blocked with 5% reconstituted skim milk powder in TBST solution (10 mM Tris–HCl pH 7.4 containing 150 mM NaCl and 0.05% Tween 20). The blots were incubated with CEBPD antibody (1:500, ab65081, Abcam, Burlingame, CA) in TBST overnight at 4°C. After washing with TBST, horseradish peroxidase-conjugated secondary antibodies (1:10 000, ab97051, Abcam, Burlingame, CA) were applied and the blots developed by the Enhanced Chemiluminescence Detection System (Pierce, ThermoFisher Scientific, Waltham, MA). Levels of beta-tubulin were used as an internal standard for equal loading.

## Supporting information

S1 Fig Active area is an alternative summary metric for dose-response curves. It has advantages relative to the more popular IC50: it is well-defined even if no growth inhibition is observed at any tested drug concentration, it is less sensitive to measurements around the IC50 point, and better represents whether there is a tail of resistance cells.
(EPS)

S2 Fig Predictive performance on CTRPv2 assessed using mean concordance index across drugs (10-fold cross-validation). (EPS)

S3 Fig Lacrosse’s performance on CTRPv2 is robust to the exact choice of the MRF strength parameter (edge weight). (EPS)

S4 Fig LOBICO [18] attempts to find small logical networks that combine mutation status to predict binarized drug response. We tested LOBICO using only mutation data from CTRPv2 and found it was consistently and severely outperformed by LASSO in terms of predictive performance. LASSO here refers to linear regression on the AUCs, whereas GLM is L1-regularized logistic regression on the same binarized response as for LOBICO. 10-fold cross-validation was used for all methods: to choose λ for the regression approaches and the model complexity for LOBICO (using the same 8 complexity settings as used in the LOBICO paper). It is possible that LOBICO would be more competitive if we used a smaller set of known important mutations. Since we are interested in discovering such relationships *de novo* we did not explore this approach further.
(EPS)

S5 Fig Lacrosse’s generalization performance in CCLE having been trained on CTRPv2. (EPS)

S6 Fig Lacrosse’s generalization performance in gCSI having been trained on CTRPv2. (EPS)

S7 Fig Lacrosse’s generalization performance in GDSC1000 having been trained on CTRPv2. (EPS)

S8 Fig Other latent characteristics discovered by Lacrosse using CCLE, showing the drugs in the LC and predictive genomic features. *p*-values are from a Spearman correlation test. LCs noted in the text are shown in Fig 3.
(EPS)

S9 Fig Comparing the global latent characteristic discovered by Lacrosse to the general level of drug sensitivity (GLDS) described by Geeleher et al. [27]. (EPS)

S10 Fig Successful knock-down of C/EBP*δ* using siRNA in the BT-549 breast cancer cell-line. **a**. By bright-field microscopy cells appear healthy/viable after knock-down. **b**. Western blot analysis confirms that C/EBP*δ* protein levels are substantially reduced following overnight (O/N) treatment with the targeting siRNA, and that this knock-down remains substantial after 24 hours.
(TIFF)
