# Supplementary figures and images for: Sparse discriminative latent characteristics for predicting cancer drug sensitivity from genomic features
Source: PLoS Comput Biol. 2019 May 28;15(5):e1006743. doi: 10.1371/journal.pcbi.1006743 (PMC6555538; doi:10.1371/journal.pcbi.1006743)

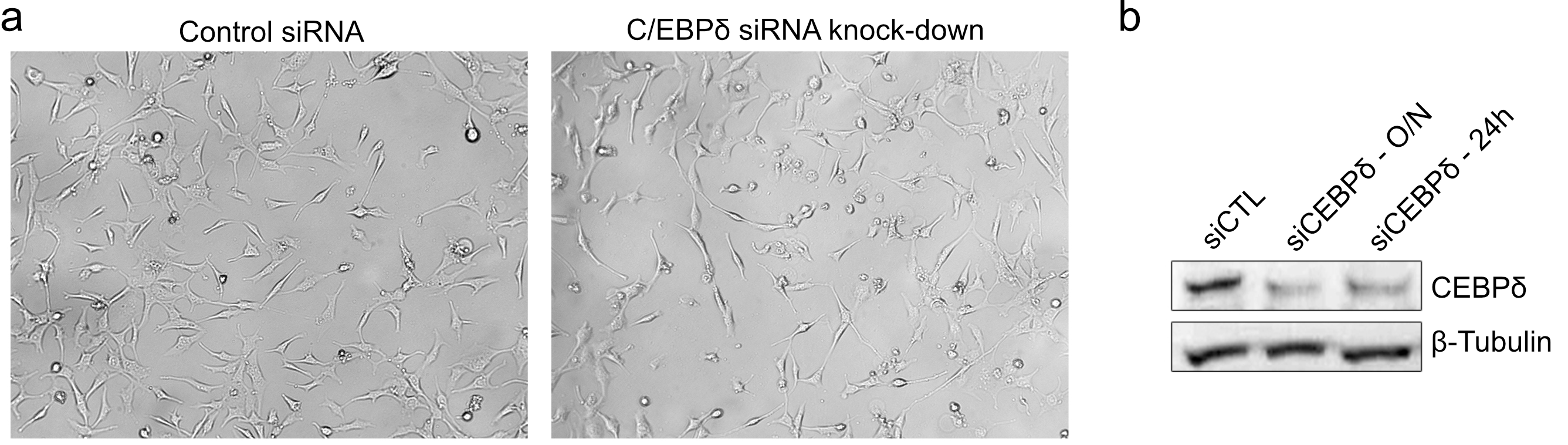

Supplement: S10 Fig — a. By bright-field microscopy cells appear healthy/viable after knock-down. b. Western blot analysis confirms that C/EBPδ protein levels are substantially reduced following overnight (O/N) treatment with the targeting siRNA, and that this knock-down remains substantial after 24 hours. (TIFF) [file pcbi.1006743.s010.tiff]
